# Complete Genome Sequence of Corynebacterium minutissimum, an Opportunistic Pathogen and the Causative Agent of Erythrasma

**DOI:** 10.1128/genomeA.00139-15

**Published:** 2015-03-19

**Authors:** Patricia K. Penton, Eishita Tyagi, Ben W. Humrighouse, John R. McQuiston

**Affiliations:** a Special Bacteriology Reference Laboratory, Bacterial Special Pathogens Branch, Division of High-Consequence Pathogens and Pathology, National Center for Emerging and Zoonotic Infectious Diseases, Centers for Disease Control and Prevention, Atlanta, Georgia, USA; b Laboratory Preparedness and Response Branch, Division of Preparedness and Emerging Infections, National Center for Emerging and Zoonotic Infectious Diseases, Centers for Disease Control and Prevention, Atlanta, Georgia, USA

## Abstract

Corynebacterium minutissimum was first isolated in 1961 from infection sites of patients presenting with erythrasma, a common cutaneous infection characterized by a rash. Since its discovery, C. minutissimum has been identified as an opportunistic pathogen in immunosuppressed cancer and HIV patients. Here, we report the whole-genome sequence of C. minutissimum.

## GENOME ANNOUNCEMENT

Corynebacterium minutissimum is a Gram-positive, non-spore-forming, facultatively anaerobic bacillus. It is the causative agent of erythrasma (1) and an opportunistic pathogen for immunosuppressed cancer and HIV patients. Corynebacterium minutissimum has been isolated from granulomatous lesions, subcutaneous abscesses, and cutaneous fistulas of two HIV-positive patients (2, 3). Bacteremia with C. minutissimum and resultant sepsis have been reported in patients with chronic and acute myeloid leukemia (2, 4). Multiantibiotic resistance has been observed with C. minutissimum infection (5, 6), and virulence factors associated with this pathogen remain ambiguous, thus highlighting the need to obtain complete genomic data. The identification of genes contributing to antibiotic resistance and pathogenicity may assist in elucidating the mechanism of infection, better treatment options, and accurate identification tools.

Corynebacterium minutissimum strain ATCC 23348 was grown on heart infusion agar supplemented with 5% rabbit blood at 35°C overnight. Genomic DNA was isolated via a phenol-chloroform-CTAB extraction as previously described (7). Genome libraries were sequenced on an Illumina HiSeq 2000 platform, generating 2 × 100 paired-end sequencing libraries with an average insert size of 945 bp.

All sequence reads were filtered and trimmed at both the 5′ and 3′ ends based on a threshold of Q30 using CG-Pipeline (8). Passed Illumina reads were assembled using SPAdes version 3.1.1 (9). This *de novo* assembly approach yielded 40 large contigs (500 bp each) with an *N*_50_ size of 207,332 bp, an average length of 65,591 bp, and a maximum length of 403,231 bp. The resulting genome sequence was 2,663,628 Mb with 200-fold average coverage and an average GC content of 59.96%. An optical map was generated for the genome using the ARGUS whole-genome optical mapping system from OpGen. The optical map assembly of the single-molecule restriction maps was generated using enzyme AflII, and resulted in a single 2,687,481-bp chromosome. The 40-contig assembly was then converted to *in silico* optical maps using the AflII enzyme and mapped to the OpGen optical map for contig ordering and finishing. This approach resulted in a final assembly of 38 contigs with a total size of 2,663,401 bp. Of these 38 contigs, 17 contigs comprising a total of 2,452,554 bp were ordered with respect to the optical map, and 21 contigs comprising a total of 210,847 bp were unordered. The final assembly had an *N*_50_ size of 293,999 bp with an average contig length of 70,089 bp and a maximum contig length of 403,231 bp.

Corynebacterium reference sequences were downloaded through NCBI and converted into *in silco* optical maps, using restriction enzyme AflII, for a preliminary unweighted-pair group with arithmetic mean (UPGMA) analysis with the whole-genome optical map of C. minutissimum strain ATCC 23348. The resulting UPGMA tree revealed close clustering of the C. minutissimum genome with the C. diptheriae and C. pseudotuberculosis reference sequences. Annotation of the final assembly using GeneMarkS (10), tRNAscan-SE 1.21 (11), and RNAmmer 1.2 (12) revealed 2,469 protein-coding regions, 51 tRNA genes, and 3 rRNA operons.

### Nucleotide sequence accession numbers.

This whole-genome shotgun project has been deposited at GenBank under the BioProject ID PRJNA264738, BioSample accession number SAMN03140311, and accession number JSEF00000000. The version described in this paper is version JSEF01000000.
